# Disordered IL-33/ST2 Activation in Decidualizing Stromal Cells Prolongs Uterine Receptivity in Women with Recurrent Pregnancy Loss

**DOI:** 10.1371/journal.pone.0052252

**Published:** 2012-12-27

**Authors:** Madhuri S. Salker, Jaya Nautiyal, Jennifer H. Steel, Zoe Webster, Sandra Šućurović, Marilena Nicou, Yogesh Singh, Emma S. Lucas, Keisuke Murakami, Yi-Wah Chan, Sean James, Yazan Abdallah, Mark Christian, B. Anne Croy, Biserka Mulac-Jericevic, Siobhan Quenby, Jan J. Brosens

**Affiliations:** 1 Division of Reproductive Health, Warwick Medical School, Clinical Sciences Research Laboratories, University Hospital, Coventry, United Kingdom; 2 Institute of Reproductive and Developmental Biology, Imperial College London, Hammersmith Campus, London, United Kingdom; 3 Embryonic Stem Cell Facility, MRC Clinical Sciences Centre, Imperial College London, Hammersmith Campus, London, United Kingdom; 4 Department of Physiology and Immunology, Medical School, University of Rijeka, Rijeka, Croatia; 5 Department of Infection and Immunity, The Royal Veterinary College, Royal College Street, London, United Kingdom; 6 Department of Biomedical and Molecular Sciences, Queen's University, Kingston, Ontario, Canada; McGill University, Canada

## Abstract

Decidualization renders the endometrium transiently receptive to an implanting blastocyst although the underlying mechanisms remain incompletely understood. Here we show that human endometrial stromal cells (HESCs) rapidly release IL-33, a key regulator of innate immune responses, upon decidualization. In parallel, differentiating HESCs upregulate the IL-33 transmembrane receptor ST2L and other pro-inflammatory mediators before mounting a profound anti-inflammatory response that includes downregulation of ST2L and increased expression of the soluble decoy receptor sST2. We demonstrate that HESCs secrete factors permissive of embryo implantation in mice only during the pro-inflammatory phase of the decidual process. IL-33 knockdown in undifferentiated HESCs was sufficient to abrogate this pro-inflammatory decidual response. Further, sequential activation of the IL-33/ST2L/sST2 axis was disordered in decidualizing HESCs from women with recurrent pregnancy loss. Signals from these cultures prolonged the implantation window but also caused subsequent pregnancy failure in mice. Thus, Il-33/ST2 activation in HESCS drives an autoinflammatory response that controls the temporal expression of receptivity genes. Failure to constrain this response predisposes to miscarriage by allowing out-of-phase implantation in an unsupportive uterine environment.

## Introduction

For pregnancy to succeed, the human endometrium must first engage with a competent embryo, embed the conceptus in decidualizing stroma, and then support deep uterine invasion of extra-embryonic trophoblast [1], [2]. These dynamic events require a carefully prepared, specialized uterine microenvironment. The preparatory process for pregnancy starts with the postovulatory surge in circulating progesterone levels, which in turn inhibits estrogen-dependent proliferation of the uterine epithelium, induces secretory transformation of the uterine glands, and recruits uterine natural killer (uNK), macrophages and other immune cells to the endometrium [3], [4], [5]. Subsequently, the luminal epithelium expresses an evolutionarily conserved repertoire of molecules essential for stable interaction and adherence of a blastocyst, thus enabling implantation [6], [7], [8]. Importantly, receptivity is a transient endometrial state, confined to only a few days in the mid-secretory phase of the cycle, and dependent on paracrine signals from stromal cells underlying the luminal epithelium [9], [10]. Failure to express a receptive phenotype is thought to be a major cause of subfertility and IVF treatment failure [11], [12]. Conversely, prolonged endometrial receptivity facilitates implantation of delayed or compromised embryos and has a strong association with early pregnancy loss [12], [13], [14]. Thus, the timing and duration of the so-called ‘window of implantation’ are major endometrial determinants of the likelihood of reproductive success.

Decidualization denotes the differentiation process by which resident endometrial stromal cells acquire a specialized secretory phenotype [15], [16]. In fact, secreted factors, such as prolactin (PRL) and insulin-like growth factor-binding protein 1 (IGFBP1), are widely used to assess the quality of the decidual response in human endometrial stromal cells (HESCs) [17]. Hence, decidualization bestows on stromal cells the ability to create paracrine gradients essential for uterine receptivity and post-implantation pregnancy support. Decidual cells also function as gatekeepers of different immune cells at the feto-maternal interface. For example, differentiating HESCs secrete interleukin 11 (IL-11) and IL-15, implicated in recruitment and differentiation of uterine natural killer (uNK) cells, which in turn are a rich source of angiogenic factors [18], [19], [20]. On the other hand, decidual cells surrounding the early conceptus have been shown to epigenetically silence key chemokine genes, thus protecting the allogeneic fetus from infiltrating cytotoxic T lymphocytes [21]. To accomplish this multitude of functions, decidual cells must adapt dynamically and tailor their secretome to accommodate the various stages of the implantation process [22]. The mechanisms that control these transient changes in the phenotype of decidual cells over the window of implantation are largely unknown. A Th1/Th2 paradigm has been used to describe the changing inflammatory endometrial microenvironment in early pregnancy [23], [24]. While a Th1-type response, characterized by induction of pro-inflammatory cytokines (e.g. IL-2, IL-12 and interferon-γ), is thought to underpin endometrial receptivity and early implantation, a preponderance of regulatory or anti-inflammatory (Th2-type) cytokines (e.g. IL-4, IL-5 and IL-10) seems essential for maintaining pregnancy [25].

IL-33, a member of the IL-1 family, is a key regulator of inflammatory and immune processes [26], [27], [28]. It was first identified as a nuclear factor in endothelial cells. Subsequently it was shown that IL-33 is a potent pro-inflammatory danger signal, or alarmin, released from necrotic cells upon trauma or infection [29]. As a nuclear factor, IL-33 may be involved in transcriptional repression and regulation of chromatin compaction by promoting nucleosome-nucleosome interactions [30]. The pro-inflammatory effects of IL-33, however, require cellular release and binding to its cell-surface receptor, consisting of ST2, a member of the family of IL-1 receptor and encoded by *IL1LR1*, and IL-1R accessory protein (IL-1RAcP) [26], [31]. There are two main ST2 isoforms: a long transmembrane form (ST2L) and a soluble decoy receptor (sST2), which lacks both transmembrane and intracellular domains [26]. ST2L is widely expressed on effector immune cells, including T lymphocytes, NK and NKT cells, eosinophils, basophils, and mast cells, enabling IL-33 to drive the host defence to pathogens and innate immune responses [32]. Secreted sST2 binds and inhibits IL-33, thus serving as a potent anti-inflammatory mediator [26].

Here we report that activation of the IL-33/ST2 pathway in decidualizing HESCs induces a self-limiting autoinflammatory response that controls the transient expression of uterine receptivity genes. We further provide evidence that the ability of HESCs to restrain this pro-inflammatory response upon decidualization is impaired in patients suffering from recurrent pregnancy loss (RPL), thus permitting embryo implantation in an unsupportive uterine environment.

## Methods

### Patient selection and sample collection

The study was approved by Hammersmith and Queen Charlotte’s & Chelsea Research Ethics Committee (1997/5065). Written informed consent was obtained from all patients before endometrial sampling. Subjects attended the clinic for conception delay (controls) or RPL and were investigated according to the standard clinical protocols. RPL was defined as three or more consecutive pregnancy losses before 24 weeks gestation. Pipelle biopsies were obtained throughout the cycle or timed to the mid-secretory phase. An ovulation prediction kit (Assure Ovulation Predictor, San Diego, CA, USA) was used to obtain samples 6 to 10 days after the pre-ovulatory LH surge (LH+6 to LH+10).

### Primary HESC Cultures

HESCs were isolated from endometrial tissues as previously described [33]. Purified HESCs were expanded in maintenance medium of DMEM/F-12 containing 10% dextran-coated charcoal-treated fetal bovine serum (DCC-FBS) and 1% antibiotic-antimycotic solution. Confluent monolayers were decidualized in DMEM/F-12 containing 2% DCC-FBS with 0.5 mM 8-bromo-cAMP (8-br-cAMP; Sigma, Poole, UK) with or without 10^–6 ^M medroxyprogesterone acetate (MPA; Sigma) to induce a differentiated phenotype. Recombinant human IL-33 was used at 0.1 ng/ml (R&D Systems, UK). All experiments were carried out before the third cell passage.

### Animal Experiments

C57BL/6 mice were purchased from Charles River Ltd (Margate, UK) and all experiments were carried out in accordance with the UK Home Office regulations (PPL70/6867). To prime the uterus, immature female mice were given hormonal treatment, consisting of 10 mg/kg/day β-estradiol (Sigma, UK) for three consecutive days followed by a single dose of 1 mg progesterone (Sigma, UK). The animals were then anaesthetized, subjected to laparotomy, and both uterine horns were injected over 5 min with 50 µl of culture supernatant from undifferentiated or decidualized primary HESCs or control (unconditioned) medium. The cervix was not clamped. The incision was then closed and the animals were euthanized 24 h later. The uterine horns were dissected and either fixed in formalin or snap-frozen and stored at -80°C. To assess implantation, C57BL/6 female mice were mated with sterile males and the day of the appearance of the vaginal plug designated as 0 days post coitus (d.p.c.). Laparotomy was performed 2.5 d.p.c. Both uterine horns were injected with conditioned or unconditioned culture medium, as described above. After 10 minutes, 10 blastocysts (equivalent of 3.5 d.p.c.) were transferred to a single treated uterine horn. The uteri were harvested 3 or 6 days following surgery, ipsilateral implantation sites counted, and tissues fixed in formalin or snap-frozen for further analysis.

### Real-time Quantitative (qRT)-PCR and PCR Arrays

Total RNA was extracted from HESC cultures using Stat60 (Tel-Test, Friendswood, TX, USA). The quality of the RNA was evaluated using a Bioanalyzer 2100 (Agilent Technologies Inc., Santa Clara, CA, USA). Equal amounts of total RNA (2 µg) were reverse transcribed by using the Superscript First-Strand synthesis system for RT-PCR (Invitrogen) and the resulting cDNA used as template in qRT-PCR analysis. The gene-specific primer pairs, designed by using the Primer3 software, are available on request. L19 and Cyclophilin represent non-regulated human and murine genes, respectively, and their expression was used to normalize for variances in input cDNA. Detection of gene expression was performed with JumpStart-SYBR Green (Sigma) and the ABI stepONE Plus sequence detection system (Applied Biosystems, Foster City, CA, USA). The expression levels of the samples were expressed as arbitrary units defined by the ^ΔΔ^C_T_ method. All measurements were performed in triplicate. Melting curve analysis and agarose gel electrophoresis confirmed amplification specificity.

The RT^2^ Profiler Human Inflammatory mediators and receptors PCR Array (SA Biosciences), containing 84 genes related to the inflammatory pathway, plus housekeeping genes and controls, was used to analyze immune-related gene expression in HESCs. 2 µg of total RNA was reverse-transcribed with the First Strand kit (SA Biosciences), combined with the SYBR Green/ROX PCR master mix (SA Biosciences), and added to each well of the RT^2^ Profiler PCR plate, containing the pre-dispensed gene-specific primer sets. The reaction was run on an ABI- stepONE Plus. Data analysis was based on the C_T_ method, with normalization to four different housekeeping genes. Measurements were made in triplicate on primary cultures from 3 subjects.

### Transfection and Propidium Iodide Staining

Primary HESCs were transfected with small interfering RNA (siRNA) by the calcium phosphate co-precipitation method using the Profection mammalian transfection kit (Promega, Madison, WI), as previously described [33]. For gene silencing, undifferentiated HESCs were transiently transfected with 50 nM of the following siRNA reagents IL1RL1-siGENOME SMARTpool, IL-33-siGENOME SMARTpool, or siCONTROL Non-Targeting purchased from Dharmacon (Lafayette, CO, USA). Parallel cultures were harvested in either RIPA buffer for Western blot analysis or washed with PBS, fixed with cold 70% ethanol, stained with propidium iodide (Sigma), ribonuclease-A (Qiagen, Germany) treated and subjected to flow cytometry analysis. The degree of apoptosis in primary cultures was evaluated by measuring the sub-G1 fraction (<2N). Transfection studies were performed in triplicate and repeated on primary cultures from 3 subjects.

### Western Blot Analysis

Whole cell protein extracts were prepared by lysing cells in RIPA buffer. Protein yield was quantified using the Bio-Rad DC protein assay kit (Bio-Rad, USA). Equal amounts of protein were separated by 10% SDS-Polyacrylamide Gel Electrophoresis (SDS-PAGE) before wet-transfer onto PVDF membrane (Amersham Biosciences, UK). Nonspecific binding sites were blocked by overnight incubation with 5% nonfat dry milk in Tris-buffered saline with 1% Tween (TBS-T; 130 mmol/L NaCl, 20 mmol/L Tris, pH7.6 and 1% Tween) as previously described [33]. The following primary antibodies were used: anti-ST2 and anti-IL-33 Abcam (Cambridge, UK), anti-PARP cleavage site (214/215) from BioSource International (Camarillo, CA). The primary antibodies were used at 1∶1000 except for the antibodies to β-actin (Abcam), which was diluted 1∶100,000. Protein complexes were visualized with a chemiluminescent detection kit (GE Healthcare, UK).

### Enzyme-linked Immunosorbent Assay (ELISA) for Soluble ST2 and IL-33

Soluble ST2 and IL-33 levels in the HESC culture media were determined using an amplified two-step sandwich-type immunoassay (DuoSet®; R&D Systems, USA) according to the manufacturer’s protocol. Results were derived using the standard curve method.

### Tissue Microarray and Confocal Microscopy

Needle cores (1mm in diameter and 0.4 cm in depth) of the superficial endometrial layer were obtained from formalin-fixed and paraffin-embedded timed (LH+5 to +8) endometrial specimens (n  = 10) these were arrayed into a recipient block to construct a tissue microarray. Three µm sections were stained for IL-33 (1∶800; Sigma), as previously described [34]. Stomach was used as a positive control and the primary antibody was omitted as a negative control. Immunostaining was visualized using the MIRAX Viewer (Zeiss, Germany). Immunocytochemistry was performed on primary HESCs cultured on chamber slides. The cells were either untreated or treated with 8-br-cAMP for a total of 12 h (Sigma, UK). Cells were fixed in 4% para-formaldehyde and permeabilized with 0.5% Triton and then incubated overnight at 4°C in a humidified chamber with an anti-IL33 antibody (1∶400, Sigma). The secondary antibody used was Alexa Fluor® 488 (1∶200; Invitrogen). Slides were mounted with proGOLD (Invitrogen) and stained with 4',6-diamidino-2-phenylindole (DAPI) to visualize nuclei. Images were captured using a Leica SP5 II confocal microscope.

### Cell Viability Assays

Cultured HESCs were seeded in 96-well black plates with clear bases and maintained in 10% DCC/DMEM until confluency. Cells where transfected with or without siRNA targeting IL-33 or ST2 and then subsequently decidualized or left untreated for a total of 4 days. Cell viability was evaluated using the CellTiter 96® AQueous Non-Radioactive Cell Proliferation Assay (MTS) (Promega) according to the manufacturer's instructions.

### Statistical Analysis

Data were analyzed with the statistical package Graphpad Prism (Graphpad software Inc). Pearson's chi-square test, Student’s *t*-test and Mann-Whitney U test were used when appropriate. Logarithmic transformations were used when data were not normally distributed. Statistical significance was assumed when *P*<0.05.

## Results

### The IL-33/ST2L/sST2 Axis in Decidualizing HESCs

**Figure 1:**
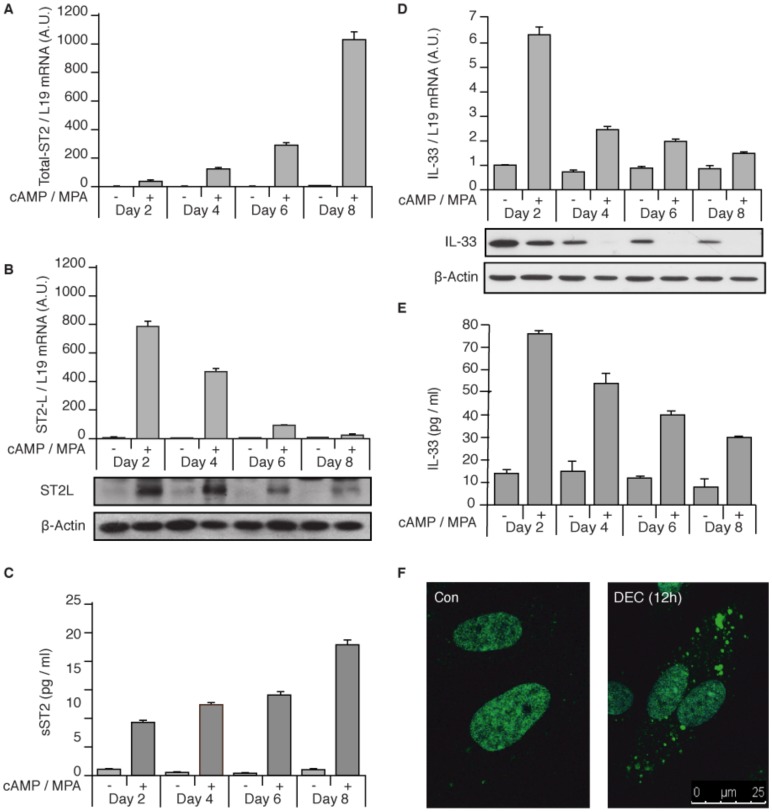
The IL-33/ST2L/sST2 axis in decidualizing HESCs. (*A*) Total ST2 transcript levels were measured in primary HESC cultures treated with 8-bromo-cAMP (cAMP) and MPA for the indicated time-points. (*B*) ST2L mRNA levels, expressed in arbitrary units (A.U.), were determined in primary cultures decidualized for 2–8 days (upper panel). Total cell lysates from parallel cultures were probed for ST2L expression (lower panel). β-actin served as a loading control. (*C*) The culture supernatant was harvested every 48 h and the levels of accumulated sST2 determined by ELISA. (*D*) Induction and release of IL-33 in decidualizing cells. Il-33 mRNA and protein was readily detectable in undifferentiated HESCs. IL-33 mRNA levels increased rapidly in response to cAMP and MPA (upper panel) but total cellular protein levels gradually declined upon decidualization (lower panel). (*E*) Il-33 rapidly accumulated in culture supernatant in response to cAMP and MPA signalling (*F*) HESCs cultured on chamber slides were decidualized for 12 h and stained for IL-33 expression (green). The nuclei were visualized with DAPI (not shown). The overlayed images were captured by confocal microscopy. Scale bar: 25 µm. Quantitative data represent the mean (± SD) of biologically triplicate experiments.

**Figure 2:**
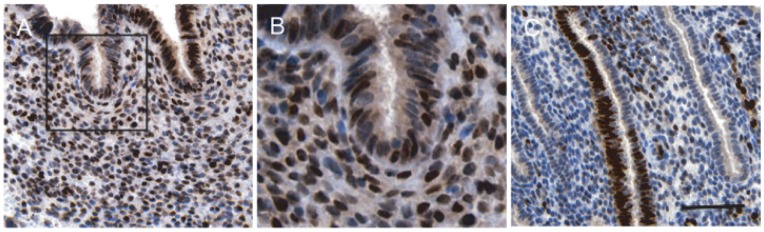
Representative IL-33 expression in mid-secretory human endometrium. Tissue sections were deparaffinised, rehydrated and stained with IL-33-specific antibody. A total of 12 biopsies from control subjects were analyzed. (*A*) Strong nuclear IL-33 immunoreactivity was observed in both the stromal and epithelial cell compartment (original magnification ×20). (*B*) Higher (×50) magnification of the indicated area also shows diffuse cytoplasmic IL-33 staining, especially near the luminal epithelium (*C*) In some specimens, IL-33 staining was very heterogeneous. For example, neighboring epithelial cells with a gland could be intensely positive and negative for IL-33 expression. (Scale Bar: 100 µm).

*IL1RL1* was identified by microarray analysis as being highly induced in HESCs decidualized with 8-br-cAMP and the progestin medroxyprogesterone acetate (MPA) for 72 h [35]. It was also found expressed in baboon endometrium during the implantation window [36]. To explore the function of this receptor in the decidual process, we first designed primers to amplify all ST2 transcripts or selectively ST2L mRNA (Fig. S1). Interestingly, while decidualization triggered a marked and time-dependent increase in total ST2 transcripts (Fig. 1A), ST2L mRNA levels followed a bi-phasic pattern, characterized by strong induction by 2 days of treatment with 8-br-cAMP and MPA followed by a gradual decline (Fig. 1B, upper panel). This pattern of ST2L mRNA regulation was mimicked at protein level (Fig. 1B, lower panel). Analysis of culture supernatants showed that HESCs secrete increasing amounts of sST2 as the decidual process proceeds (Fig. 1C). We next tested if HESCs also express IL-33. Strikingly, the expression of IL-33 transcripts in decidualizing HESCs paralleled that of ST2L with levels peaking at 2 days of differentiation (Fig. 1D, upper panel). Western blot analysis demonstrated that IL-33 is abundantly present in undifferentiated HESCs yet cellular levels decline rapidly upon decidualization (Fig. 1D, lower panel). IL-33 levels in culture supernatant increased 7-fold within 2 days decidualization, indicating that this inflammatory cytokine/alarmin is actively released by HESCs in response to cAMP and MPA signalling (Fig. 1E). In support, confocal microscopy confirmed that IL-33 is almost exclusively nuclear in undifferentiated cells. Exposure to a decidualizing signal for 12 h was sufficient to induce IL-33 cytoplasmic foci, suggesting active secretion (Fig. 1F). Tissue microarray analysis revealed intense but heterogeneous nuclear IL-33 staining in both epithelial and stromal cells in mid-secretory endometrium, although there was evidence of cytoplasmic immunoreactivity in decidualizing stromal cells underlying the lumen (Fig. 2). Together, the data show that differentiating HESCs in parallel up-regulate ST2L expression and release IL-33 before secreting increasing amounts of sST2.

**Figure 3:**
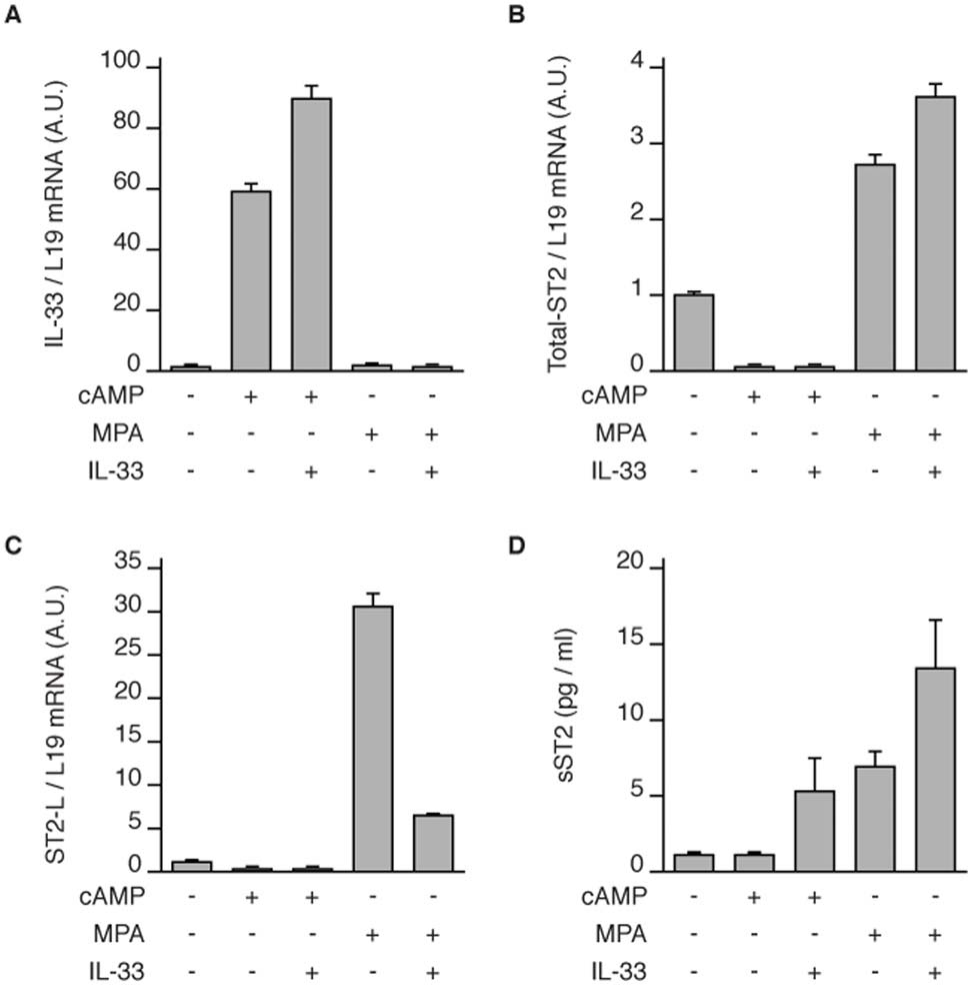
Induction of ST2 and IL-33 expression is differentially regulated. Primary HESC cultures were treated with either 8-br-cAMP or MPA, in the presence or absence of recombinant IL-33, for 72 hours and (**A-C**) IL-33, total ST2 and ST2L transcript levels were measured. (**D**) Secreted ST2 levels accumulated over 72h were measured using an ELISA. The data represent the mean (± SD) of biologically triplicate experiments primary cultures established from 3 different biopsies.

Convergence of the cAMP and progesterone pathways drives decidualization of HESCs [5]. To examine how these distinct pathways impact on the IL-33/ST2L/sST2 axis, primary cultures were treated for 72 h with 8-br-cAMP or MPA in the presence or absence of recombinant IL-33. While cAMP signalling stimulated IL-33 expression, it blunted basal ST2 mRNA levels (Fig. 3A & B). Recombinant IL-33 modestly enhanced the induction of IL-33 transcripts in response to 8-br-cAMP (Fig. 3A). Conversely, MPA did not impact on IL-33 levels in HESCs but stimulated expression of ST2 transcripts (Fig. 3B & C). Interestingly, recombinant IL-33 promoted sST2 secretion at the expense of ST2L mRNA expression (Fig. 3C & D). Thus, cAMP-dependent IL-33 expression cooperates with progestins to mount an anti-inflammatory response, exemplified by up- and down-regulation of sST2 and ST2L, respectively.

### A Transient pro-inflammatory Decidual Response Confers Endometrial Receptivity

**Figure 4:**
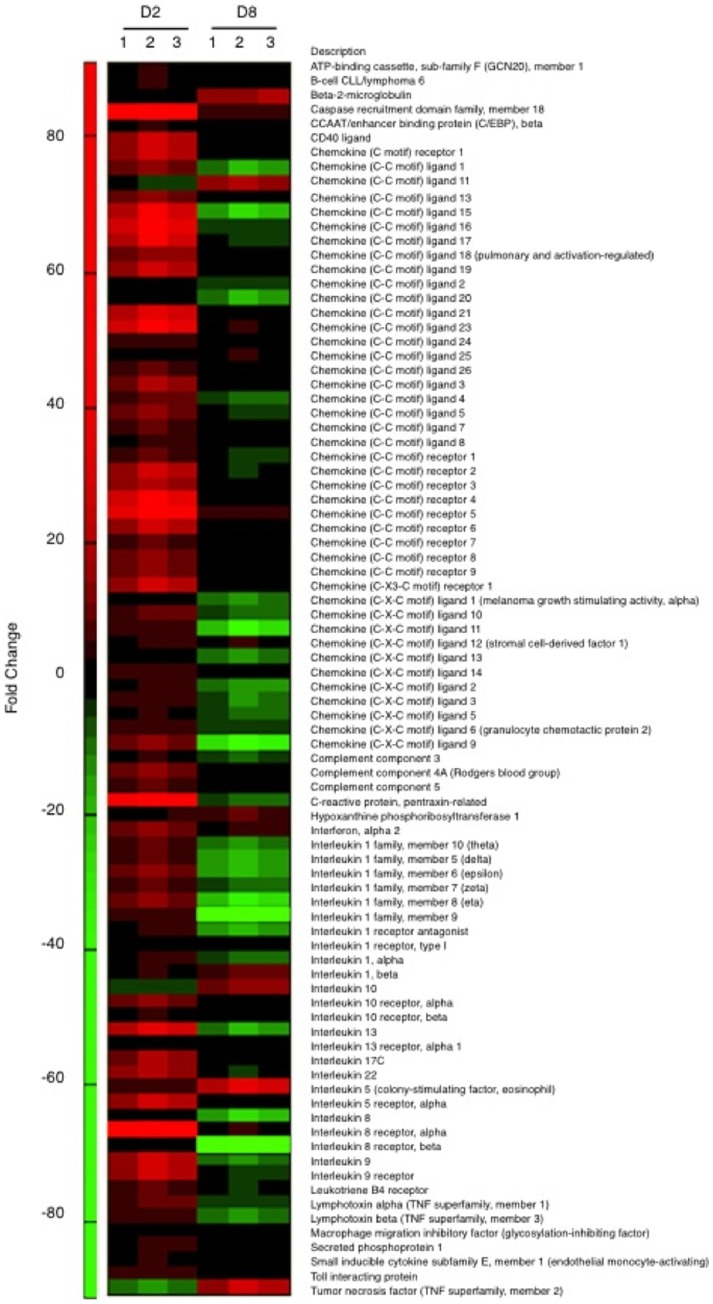
Heat-map representation of the relative change in transcripts encoding inflammatory mediators upon treatment with 8-Bromo-cAMP and MPA for 2 or 8 days (D2 or D8, respectively). Fold-change is relative to transcript levels in undifferentiated cells. Primary cultures were established from 3 different biopsies.

The pattern of IL-33 induction in decidualizing HESCs paralleled that of PROK1, a pro-inflammatory implantation cytokine [13]. We postulated that bi-phasic regulation could extend to other inflammatory mediators expressed by differentiating HESCs. To test this hypothesis, we used a qRT-PCR array to measure the expression of 84 chemokines, cytokines, interleukins, and their receptors in undifferentiated and decidualizing cultures. Treatment with 8-br-cAMP and MPA for 2 days was sufficient to significantly up-regulate 70 inflammatory mediators (>1.5-fold, *P*<0.01; Fig. 4 & Table 1). At this cut-off, only 3 transcripts (*CCL11*, *IL-10*, and *TNF-α*) were downregulated at this stage of the decidual process. By 8 days of differentiation, 12 transcripts remained elevated but 34 were now lower compared to their expression in undifferentiated cells (Table 1 & Fig. S2A). Thus IL-33 release and induction of its trans-membrane receptor in HESCs is part of a substantive but transient differentiation-dependent pro-inflammatory response, involving many chemokines, interleukins, and other mediators.

**Table 1:** Inflammatory mediators regulated upon decidualization in HESCs.

8UniGene	Interleukins & Receptors	Fold-change	
		D2	D8
Hs.306974	Interleukin 1 family, member 10 (theta)	4.66	-6.81
Hs.516301	Interleukin 1 family, member 5 (delta)	4.48	-8.75
Hs.278910	Interleukin 1 family, member 6 (epsilon)	6.16	-8.56
Hs.166371	Interleukin 1 family, member 7 (zeta)	4.26	-4.69
Hs.278909	Interleukin 1 family, member 8 (eta)	7.02	-12.53
Hs.211238	Interleukin 1 family, member 9	3.00	-42.93
Hs.1722	Interleukin 1, alpha	2.63	-5.33
Hs.126256	Interleukin 1, beta	2.36	5.02
Hs.2247	Interleukin 5	3.06	12.33
Hs.624	Interleukin 8	-1.02	-9.54
Hs.960	Interleukin 9	10.79	-6.34
Hs.193717	Interleukin 10	-3.05	7.03
Hs.845	Interleukin 13	13.25	-7.87
Hs.278911	Interleukin 17C	8.39	-1.72
Hs.287369	Interleukin 22	9.06	-2.09
Hs.81134	Interleukin 1 receptor antagonist	2.43	-8.77
Hs.701982	Interleukin 1 receptor, type I	1.33	-1.06
Hs.68876	Interleukin 5 receptor, alpha	10.44	1.38
Hs.194778	Interleukin 8 receptor, alpha	30.47	2.03
Hs.846	Interleukin 8 receptor, beta	1.16	-58.15
Hs.406228	Interleukin 9 receptor	10.73	-2.31
Hs.504035	Interleukin 10 receptor, alpha	5.99	-1.65
Hs.654593	Interleukin 10 receptor, beta	2.17	1.59
Hs.496646	Interleukin 13 receptor, alpha 1	1.74	-1.37
**UniGene**	**Chemokines & Receptors**	**D2**	**D8**
Hs.72918	Chemokine (C-C motif) ligand 1	6.59	-7.38
Hs.54460	Chemokine (C-C motif) ligand 11	-2.69	8.69
Hs.414629	Chemokine (C-C motif) ligand 13	6.72	-1.03
Hs.272493	Chemokine (C-C motif) ligand 15	13.47	-9.50
Hs.10458	Chemokine (C-C motif) ligand 16	15.76	-3.58
Hs.546294	Chemokine (C-C motif) ligand 17	13.68	-2.60
Hs.143961	Chemokine (C-C motif) ligand 18	7.34	-1.37
Hs.50002	Chemokine (C-C motif) ligand 19	9.87	1.66
Hs.303649	Chemokine (C-C motif) ligand 2	1.22	-3.21
Hs.75498	Chemokine (C-C motif) ligand 20	1.86	-8.17
Hs.57907	Chemokine (C-C motif) ligand 21	13.15	-1.44
Hs.169191	Chemokine (C-C motif) ligand 23	16.58	2.20
Hs.247838	Chemokine (C-C motif) ligand 24	3.26	-1.45
Hs.310511	Chemokine (C-C motif) ligand 25	1.89	2.15
Hs.131342	Chemokine (C-C motif) ligand 26	4.15	1.24
Hs.514107	Chemokine (C-C motif) ligand 3	7.97	-1.04
Hs.75703	Chemokine (C-C motif) ligand 4	5.25	-5.43
Hs.514821	Chemokine (C-C motif) ligand 5	6.79	-2.38
Hs.251526	Chemokine (C-C motif) ligand 7	3.90	-1.73
Hs.271387	Chemokine (C-C motif) ligand 8	2.83	-1.01
Hs.632586	Chemokine (C-X-C motif) ligand 10	4.97	-5.31
Hs.632592	Chemokine (C-X-C motif) ligand 11	2.91	-12.83
Hs.522891	Chemokine (C-X-C motif) ligand 12	2.44	2.06
Hs.100431	Chemokine (C-X-C motif) ligand 13	1.53	-6.48
Hs.483444	Chemokine (C-X-C motif) ligand 14	3.08	-1.35
Hs.590921	Chemokine (C-X-C motif) ligand 2	2.72	-7.13
Hs.89690	Chemokine (C-X-C motif) ligand 3	3.23	-5.57
Hs.89714	Chemokine (C-X-C motif) ligand 5	2.23	-4.64
Hs.164021	Chemokine (C-X-C motif) ligand 6	3.16	-3.15
Hs.77367	Chemokine (C-X-C motif) ligand 9	6.21	-14.83
Hs.248116	Chemokine (C motif) receptor 1	10.51	1.17
Hs.78913	Chemokine (C-X3-C motif) receptor 1	10.36	1.14
Hs.789	Chemokine (C-X-C motif) ligand 1	1.85	-5.99
Hs.301921	Chemokine (C-C motif) receptor 1	4.04	-2.58
Hs.511794	Chemokine (C-C motif) receptor 2	10.40	-2.26
Hs.506190	Chemokine (C-C motif) receptor 3	9.44	-1.52
Hs.184926	Chemokine (C-C motif) receptor 4	15.83	-1.45
Hs.450802	Chemokine (C-C motif) receptor 5	18.49	3.16
Hs.46468	Chemokine (C-C motif) receptor 6	9.62	-1.80
Hs.370036	Chemokine (C-C motif) receptor 7	4.70	1.48
Hs.113222	Chemokine (C-C motif) receptor 8	6.07	-1.03
Hs.225946	Chemokine (C-C motif) receptor 9	6.39	-1.67
**UniGene**	**Miscellaneous**	**D2**	**D8**
Hs.655285	ATP-binding cassette, sub-family F (GCN20), member 1	2.21	-1.07
Hs.478588	B-cell CLL/lymphoma 6	2.35	1.48
Hs.534255	Beta-2-microglobulin	-1.49	11.13
Hs.709456	C-reactive protein, pentraxin-related	21.18	-4.63
Hs.56279	Caspase recruitment domain family, member 18	32.50	3.49
Hs.517106	CCAAT/enhancer binding protein (C/EBP), beta	1.90	-1.03
Hs.592244	CD40 ligand	10.95	1.26
Hs.529053	Complement component 3	2.11	-4.06
Hs.534847	Complement component 4A (Rodgers blood group)	6.51	-1.29
Hs.494997	Complement component 5	4.72	1.17
Hs.412707	Hypoxanthine phosphoribosyltransferase 1	2.26	3.29
Hs.211575	Interferon, alpha 2	6.20	2.44
Hs.655431	Leukotriene B4 receptor	4.08	-1.83
Hs.36	Lymphotoxin alpha (TNF superfamily, member 1)	5.41	-2.93
Hs.376208	Lymphotoxin beta (TNF superfamily, member 3)	3.17	-6.90
Hs.407995	Macrophage migration inhibitory factor	1.06	-1.17
Hs.313	Secreted phosphoprotein 1	2.59	1.57
Hs.591680	Small inducible cytokine subfamily E, member 1	2.09	-1.26
Hs.368527	Toll interacting protein	3.19	1.01
Hs.241570	Tumor necrosis factor (TNF superfamily, member 2)	-6.33	9.84

**Figure 5:**
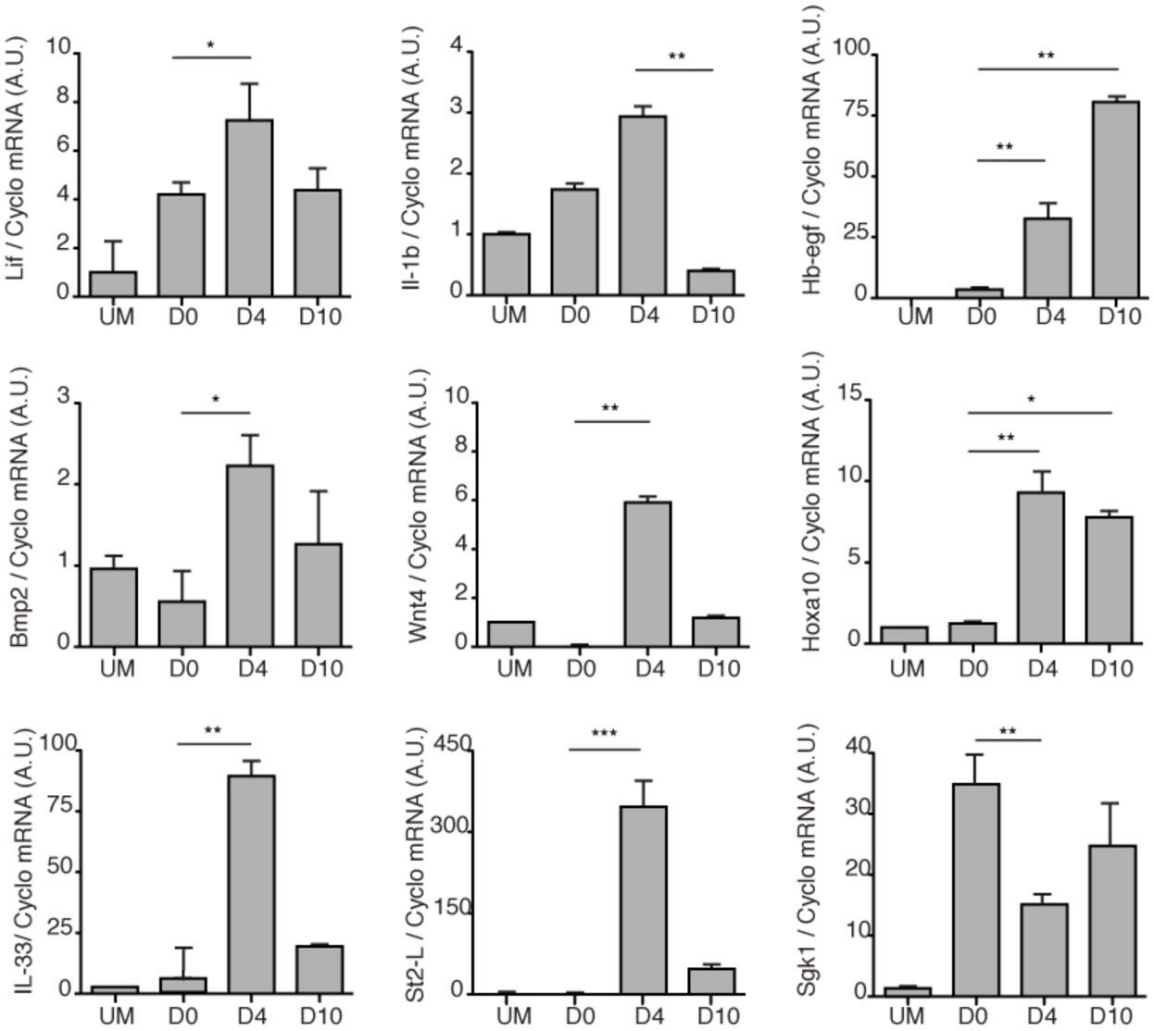
Expression of receptivity genes in the mouse uterus in response to HESC signals. The uterine horns of hormonally primed C57B/6 female mice were injected with either unconditioned medium (UM), culture supernatant from undifferentiated HESCs (D0), or from cultures decidualized for 4 or 8 days (D4 and D10, respectively). qRT-PCR was used to determine uterine expression 24 h later of the following transcripts: leukemia inhibitory factor (*Lif*), interleukin 1-β (*Il-1β*), heparin binding EGF (*Hb-egf*), bone morphogenetic protein 2 (*Bmp2*), wingless-related MMTV integration site 4 (*Wnt4*), homeobox protein 10 (*Hoxa10*), Il-33, trans-membrane ST2 (*St2l*) and serum- and glucocorticoid-inducible kinase 1 (*Sgk1*). Transcript levels were normalized to the levels of Cyclophilin-B (*Cyclo*) mRNA and expressed in arbitrary units (A.U.). The results represent mean expression (± SD). A total of 6 uterine horns were analyzed in each treatment group. *P<0.05; **P<0.01; ***P<0.001.

We speculated that soluble factors secreted by stromal cells during the pro-inflammatory phase of the decidual process could serve as signals that render the endometrium receptive. To test this hypothesis, the conditioned medium of primary HESC cultures decidualized with 8-br-cAMP and MPA was harvested every 48 h over 10 days. The uterine lumen of immature C57BL/6 female mice, primed with progesterone and estradiol, was then flushed at laparotomy with unconditioned medium, or with culture supernatant from undifferentiated HESCs, or from cells decidualized for either 4 or 10 days. The animals were sacrificed 24 h later and the expression of a panel of uterine receptivity genes examined by qRT-PCR analysis. Soluble factors accumulated in the supernatant of primary cultures decidualized for 4 days up-regulated in concert *Lif*, *Bmp2*, *Il-1β*, *Wnt4, Hb-egf,* and *Hoxa10* in murine endometrium (Fig. 5). This response was accompanied by a strong increase in *Il-33* and *St2l* transcripts. With the exception of *Hb-egf* and *Hoxa10,* the ability of cultured HESCs to generate paracrine signals that activate receptivity genes had been reduced or lost by 10 days of decidualization. Inhibition of *Sgk1* expression and activity in luminal epithelium is another important feature of the window of implantation [37]. Conditioned medium from undifferentiated HESCs strongly up-regulated mouse uterine *Sgk1* mRNA levels whereas this induction was less pronounced in response to signals from HESCs decidualized for 4 days (Fig. 5).

**Figure 6:**
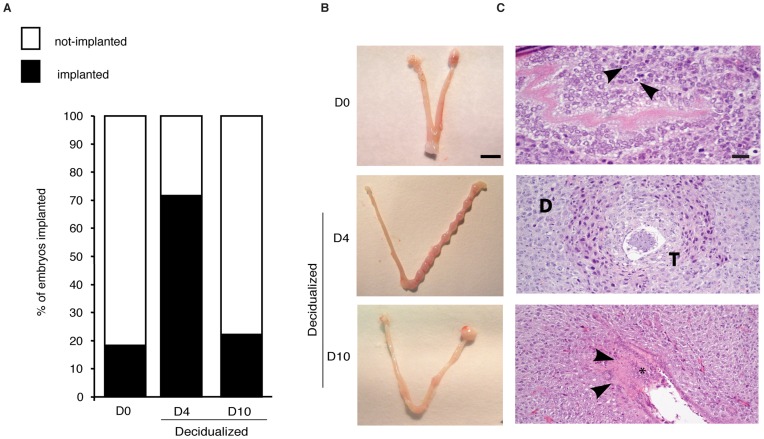
Decidualizing HESCs transiently produce factors permissive of embryo implantation in the mouse uterus. (*A*) Sterile mated C57BL/6 female mice were subjected to laparotomy and both uterine horns gently flushed with culture supernatant from undifferentiated HESCs (D0) or from cultures decidualized for 4 or 10 days (D4 and D10, respectively). Ten embryos (equivalent to stage 3.5 d.p.c.) were then transferred to the right horn. Each treatment group consisted of 6 animals. The number of implantation sites in the right horn was determined 6.5 d.p.c. Implantation rate was significantly higher after exposure to signals from D4 compared to D10 or D0 HESCs cells (*P*<0.001). (*B*) Macroscopic appearance of uteri at 6.5 d.p.c. (Scale bar; 1 cm). (*C*) H&E staining of representative implantation sites. Pathological features such as a poor decidual response with pyknotic and karyorrhectic stromal cell nuclei (arrow heads) were often seen at implantation sites following exposure to soluble factors secreted by D0 HESCs cells. Implantation sites in uteri pretreated with conditioned medium of D4 HESCs appeared normal, showing trophoblast (T) invasion and development of large decidual (D) cells. Epithelial cells were also absent from these implantation sites. Petecchia and focal bleeding (arrows heads) and leukocyte infiltrations (*) were seen at suspect implant sites in uteri pretreated with D10 HESC supernatants. These sites often showed a luminal epithelium and small, non-decidualized stromal cells, suggestive of failed implantation. (Scale bar: 100 µm).

To test if decidualizing HESCs control embryo implantation the uterine flushing experiments were repeated with pseudopregnant female mice mated with sterile males 2.5 days prior to surgery. Approximately 10 min after luminal injection of HESC conditioned medium, 10 cultured blastocysts [equivalent of 3.5 days post coitus (d.p.c)] were transferred into a single uterine horn. The number of implantation sites was determined 72 h later (equivalent of 6.5 d.p.c). Of the 60 embryos transferred in each treatment group, 42 (70%) and 45 (75%) embryos failed to implant upon prior exposure of the uterine lumen to signals from undifferentiated HESCs or cells decidualized for 10 days, respectively (Fig. 6A & B). This contrasted to 43 (72%) successful implantations after flushing with conditioned medium of HESCs differentiated with 8-br-cAMP and MPA for 4 days. Thus, decidualizing HESCs transiently produce factors conducive of pregnancy in this interspecies model. Further, several pathological features were noted following exposure of the uterine lumen to secreted factors from undifferentiated HESCs or cells decidualized for 10 d, including focal bleeding and immune cell infiltration around the conceptus (Fig. 6C). In contrast, the implantation sites following exposure to soluble signals from day 4 decidual cells appeared histologically normal.

### Autocrine IL-33 Signaling in HESCs Facilitates Embryo Implantation

**Figure 7:**
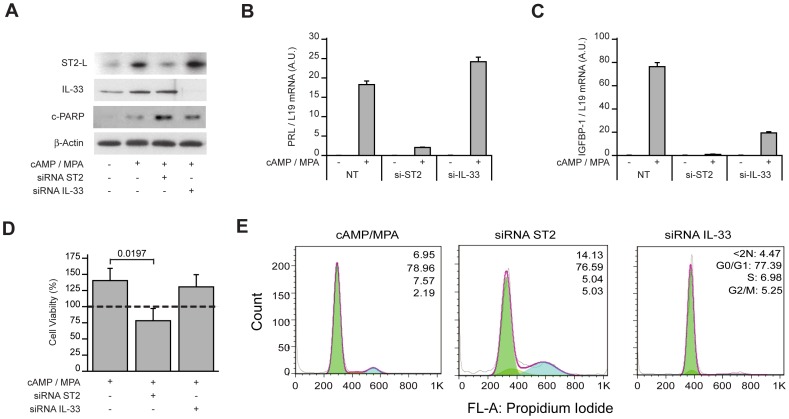
ST2 silencing in decidualizing HESCs causes cell death. (*A*) IL-33 knockdown in HESCs. Confluent primary cultures were transfected first with non-targeting, ST2, or IL-33 siRNA and then treated with 8-br-cAMP and MPA for 72 h. Whole-cell lysates were immunoprobed for anti-ST2L, anti-IL-33 and cleaved-poly (ADP-ribose) polymerase (c-PARP). β-actin served as a loading control. (*B*) Total RNA obtained from parallel cultures was subjected to qRT-PCR analysis. ST2 but not IL-33 knockdown inhibited the expression of decidual PRL mRNA. (*C*) In contrast, ST2 as well as IL-33 knockdown inhibited the induction of IGFBP1 transcripts in decidualizing cell. (*D*) The viability of HESCs, transfected first with non-targeting (NT) siRNA, IL-33 or ST2 targeting siRNA and then decidualized, was measured and expressed relatively (%) to the number of viable cells in mock-transfected, undifferentiated cells (dotted line). (*E*) The percentage of dead or dying cells (<2N;) and viable cells (G0/G1, S, and G2/M) was determined by flow cytometry of ethanol-fixed propidium iodide-stained cells. Results are representative of 3 independent experiments. The data represent the mean (± SD) of primary cultures established from 3 different biopsies.biologically triplicate experiments.

**Figure 8:**
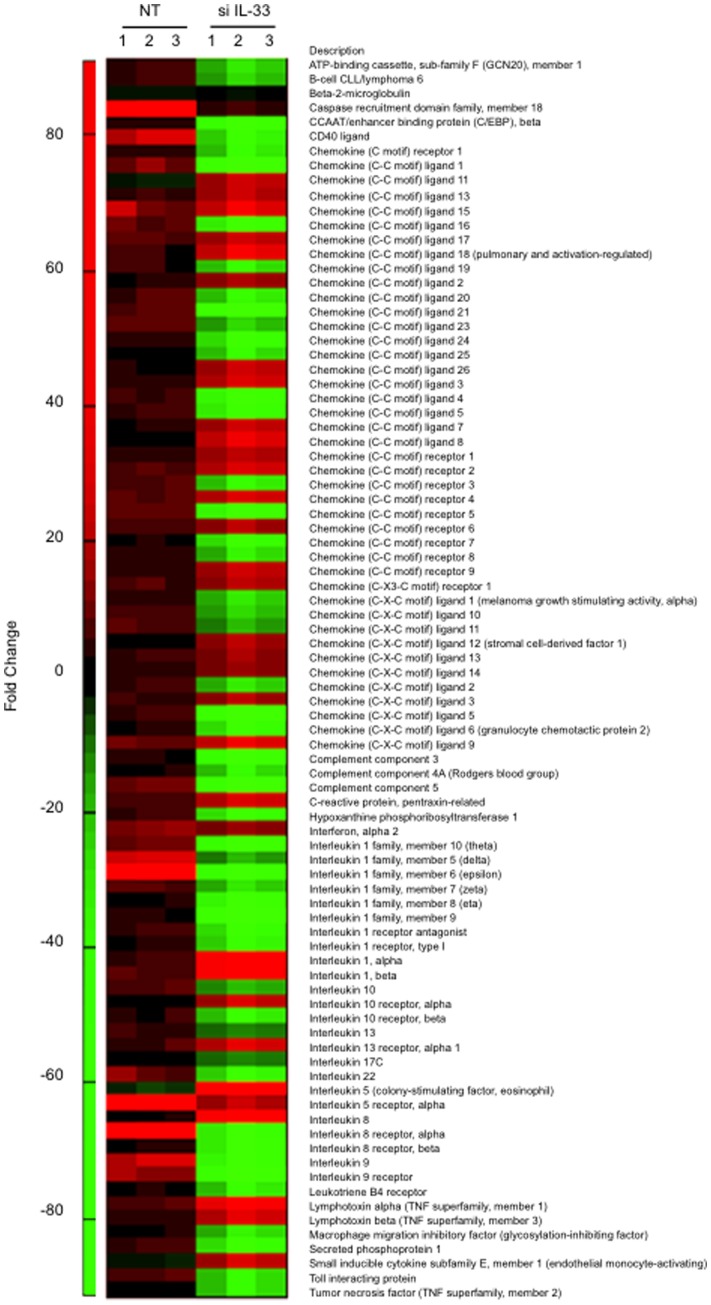
Heat-map representation of the altered expression of decidual inflammatory mediators following IL-33 knockdown. Three independent primary cultures were transfected with non-targeting (NT) or IL-33 siRNA (si IL-33) prior to treatment with 8-br-cAMP and MPA for 2 days. Fold-change is relative to transcript levels in undifferentiated cells. Primary cultures were established from 3 different biopsies.

The simultaneous induction of ST2L and release of stored IL-33 suggested activation of an autocrine pathway possibly involved in coordinating the expression of pro-inflammatory genes in decidualizing cells. To test this conjecture, we used siRNA to silence ST2L or IL-33 before differentiating primary HESC cultures (Fig. 7A). ST2L knockdown not only profoundly attenuated induction of decidual markers, *PRL* and *IGFBP1*, but also compromised the viability of decidualizing cells (Fig. 7B–E). IL-33 knockdown, however, selectively inhibited the *IGFBP1* expression in response to 8-br-cAMP and MPA stimulation; without impacting on cell viability (Fig. 7B–E). We therefore monitored the inflammatory decidual response only following IL-33 knockdown. While the procedure impacted on the expression of individual genes, the strong and coordinated induction of chemokines, cytokines, interleukins, and their receptors was maintained in primary cultures transfected with non-targeting oligos and treated with 8-br-cAMP and MPA for 48 h (Fig. 8 & Table 2). IL-33 silencing not only prevented the induction of many pro-inflammatory genes in decidualizing cells but actively reduced the expression of several inflammatory mediators, especially interleukins and chemokines, to levels below those present in undifferentiated HESCs (Fig. S2B).

**Table 2:** Inflammatory mediators regulated decidualizing HESCs upon transfection with non-targeting (NT) or IL-33 siRNA (si IL-33).

UniGene	Interleukins & Receptors	Fold-change	
		NT	si IL-33
Hs.306974	Interleukin 1 family, member 10 (theta)	6.24	-31.67
Hs.516301	Interleukin 1 family, member 5 (delta)	16.00	-14.22
Hs.278910	Interleukin 1 family, member 6 (epsilon)	34.25	-30.37
Hs.166371	Interleukin 1 family, member 7 (zeta)	4.86	-19.11
Hs.278909	Interleukin 1 family, member 8 (eta)	1.10	-29.43
Hs.211238	Interleukin 1 family, member 9	2.02	-35.75
Hs.1722	Interleukin 1, alpha	3.52	24.62
Hs.126256	Interleukin 1, beta	4.30	24.37
Hs.2247	Interleukin 5 (colony-stimulating factor, eosinophil)	-4.77	33.68
Hs.624	Interleukin 8	1.37	25.87
Hs.960	Interleukin 9	14.46	-28.4
Hs.193717	Interleukin 10	4.09	-15.39
Hs.845	Interleukin 13	2.81	-11.53
Hs.278911	Interleukin 17C	1.12	-11.48
Hs.287369	Interleukin 22	6.14	-23.78
Hs.81134	Interleukin 1 receptor antagonist	3.26	-24.71
Hs.701982	Interleukin 1 receptor, type I	1.78	-23.09
Hs.68876	Interleukin 5 receptor, alpha	33.98	11.73
Hs.194778	Interleukin 8 receptor, alpha	40.83	-27.25
Hs.846	Interleukin 8 receptor, beta	1.55	-27.47
Hs.406228	Interleukin 9 receptor	9.72	-33.13
Hs.504035	Interleukin 10 receptor, alpha	1.05	13.03
Hs.654593	Interleukin 10 receptor, beta	2.25	-21.98
Hs.496646	Interleukin 13 receptor, alpha 1	3.00	14.42
**UniGene**	**Chemokines & Receptors**	**NT**	**si IL-33**
Hs.72918	Chemokine (C-C motif) ligand 1	6.50	-35.29
Hs.54460	Chemokine (C-C motif) ligand 11	-3.60	12.33
Hs.414629	Chemokine (C-C motif) ligand 13	2.67	11.69
Hs.272493	Chemokine (C-C motif) ligand 15	8.72	16.70
Hs.10458	Chemokine (C-C motif) ligand 16	5.45	-27.80
Hs.546294	Chemokine (C-C motif) ligand 17	5.00	12.75
Hs.143961	Chemokine (C-C motif) ligand 18	2.86	16.81
Hs.50002	Chemokine (C-C motif) ligand 19	3.12	-21.17
Hs.303649	Chemokine (C-C motif) ligand 2	1.63	10.05
Hs.75498	Chemokine (C-C motif) ligand 20	3.83	-21.45
Hs.57907	Chemokine (C-C motif) ligand 21	5.29	-34.69
Hs.169191	Chemokine (C-C motif) ligand 23	5.55	-17.77
Hs.247838	Chemokine (C-C motif) ligand 24	1.71	-26.01
Hs.310511	Chemokine (C-C motif) ligand 25	-0.48	-21.21
Hs.131342	Chemokine (C-C motif) ligand 26	1.23	12.81
Hs.514107	Chemokine (C-C motif) ligand 3	2.68	14.06
Hs.75703	Chemokine (C-C motif) ligand 4	3.37	-29.26
Hs.514821	Chemokine (C-C motif) ligand 5	3.09	-26.73
Hs.251526	Chemokine (C-C motif) ligand 7	1.90	12.43
Hs.271387	Chemokine (C-C motif) ligand 8	1.13	15.47
Hs.789	Chemokine (C-X-C motif) ligand 1	2.48	-18.83
Hs.632586	Chemokine (C-X-C motif) ligand 10	3.33	-17.01
Hs.632592	Chemokine (C-X-C motif) ligand 11	4.57	-14.84
Hs.522891	Chemokine (C-X-C motif) ligand 12	0.38	10.43
Hs.100431	Chemokine (C-X-C motif) ligand 13	3.27	9.04
Hs.483444	Chemokine (C-X-C motif) ligand 14	2.46	8.77
Hs.590921	Chemokine (C-X-C motif) ligand 2	3.65	-19.15
Hs.89690	Chemokine (C-X-C motif) ligand 3	2.99	10.62
Hs.89714	Chemokine (C-X-C motif) ligand 5	2.98	-33.39
Hs.164021	Chemokine (C-X-C motif) ligand 6	1.90	-24.39
Hs.77367	Chemokine (C-X-C motif) ligand 9	5.99	16.60
Hs.248116	Chemokine (C motif) receptor 1	2.74	-22.37
Hs.78913	Chemokine (C-X3-C motif) receptor 1	3.55	11.06
Hs.301921	Chemokine (C-C motif) receptor 1	2.09	11.39
Hs.511794	Chemokine (C-C motif) receptor 2	3.94	13.78
Hs.506190	Chemokine (C-C motif) receptor 3	3.98	-21.46
Hs.184926	Chemokine (C-C motif) receptor 4	4.88	13.72
Hs.450802	Chemokine (C-C motif) receptor 5	5.44	-29.27
Hs.46468	Chemokine (C-C motif) receptor 6	3.89	10.43
Hs.370036	Chemokine (C-C motif) receptor 7	1.30	-24.64
Hs.113222	Chemokine (C-C motif) receptor 8	2.47	-20.50
Hs.225946	Chemokine (C-C motif) receptor 9	2.23	12.34
**UniGene**	**Miscellaneous**	**NT**	**si IL-33**
Hs.655285	ATP-binding cassette, sub-family F (GCN20)member 1	2.96	-19.04
Hs.478588	B-cell CLL/lymphoma 6	3.15	-17.29
Hs.534255	Beta-2-microglobulin	-2.00	-1.51
Hs.709456	C-reactive protein, pentraxin-related	3.18	14.05
Hs.56279	Caspase recruitment domain family, member 18	43.55	3.01
Hs.517106	CCAAT/enhancer binding protein (C/EBP), beta	2.55	-31.53
Hs.592244	CD40 ligand	14.67	-23.38
Hs.529053	Complement component 3	2.16	-31.10
Hs.534847	Complement component 4A (Rodgers blood group)	1.73	-21.12
Hs.494997	Complement component 5	6.32	-33.68
Hs.412707	Hypoxanthine phosphoribosyltransferase 1	3.03	-24.39
Hs.211575	Interferon, alpha 2	8.30	8.65
Hs.655431	Leukotriene B4 receptor	1.47	-22.35
Hs.36	Lymphotoxin alpha (TNF superfamily, member 1)	4.24	20.78
Hs.376208	Lymphotoxin beta (TNF superfamily, member 3)	2.58	14.55
Hs.407995	Macrophage migration inhibitory factor	1.42	-20.00
Hs.313	Secreted phosphoprotein 1	3.47	-24.40
Hs.591680	Small inducible cytokine subfamily E, member 1	-2.80	13.00
Hs.368527	Toll interacting protein	4.28	-21.12
Hs.241570	Tumor necrosis factor (TNF superfamily, member 2)	0.94	-21.23

**Figure 9:**
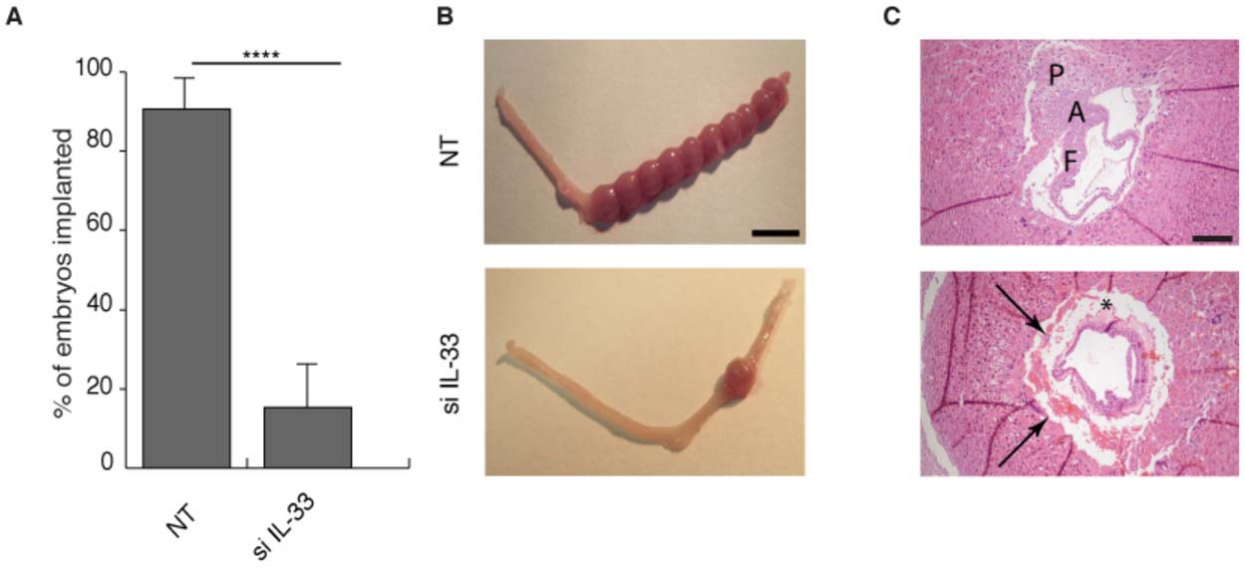
IL-33 knockdown in decidualizing HESCs impairs embryo implantation. (*A*) Vas mated C57BL/6 female mice were subjected to laparotomy and both uterine horns gently flushed with culture supernatant from primary HESC cultures first transfected with non-targeting (NT) or IL-33 siRNA (si IL-33) and then decidualized for 4 (D4) with 8-bromo-cAMP and MPA. Ten embryos (equivalent to stage 3.5 d.p.c.) were then transferred to the right horn. Each treatment group consisted of 5 animals. The number of implantation sites in the right horn was determined at 9.5 d.p.c. Implantation was dramatically reduced following exposure to culture supernatant from IL-33 deficient D4 HESCs. ***P<0.001. (*B*) Macroscopic appearance of uteri at 9.5 d.p.c. (Scale bar: 1 cm). (*C*) H&E staining of representative implantation sites. Upper panel shows a normal early pregnancy following flushing of uterine lumen culture supernatant from primary HESCs transfected with NT siRNA. Note the placental (P) primordium, fusion of allantois (A) to chorion, and fetal (F) tissue. Lower panel; exposure to culture supernatant from IL-33 deficient HESCs decidualized for 4 days (D4) resulted in an array of pathological features in early pregnancy, including focal bleeding at the feto-maternal interface (arrows). Irregularity and pinkness of the amniotic fluid suggest collapse of the amnion and coagulation of its content (*). (Scale bar: 100 µm).

We next examined the functional consequences of IL-33 knockdown in HESCs on embryo implantation in the mouse uterus. Primary HESC cultures were transfected with non-targeting oligos or IL-33 siRNA and treated with 8-br-cAMP and MPA for 4 days. Conditioned media accumulated over the last 48 h were used to flush uteri of pseudopregnant female mice prior to blastocyst transfer. IL-33 knockdown prior to decidualization of HESCs produced supernatants with dire consequences for pregnancy. These supernatants reduced the implantation rate of transferred embryos from 90% (45 implantation sites out of 50 transferred embryos) to 14% (7 sites out of 50 transferred embryos; *P*<0.001) (Fig. 9A & B). Taken together, the data show that IL-33 knockdown is sufficient to qualitatively transform the initial pro-inflammatory decidual response into an anti-inflammatory response akin to that observed upon prolonged differentiation (Fig. S2 B). Histological examination demonstrated that uterine exposure to this altered decidual inflammatory response prior to embryo transfer jeopardized the subsequent formation of a functional decidual-placental interface in the few implantation sites present 6 days later (equivalent of 9.5 d.p.c; Fig. 9C).

### The IL-33/ST2L/sST2 Axis is Deregulated in Decidualizing HESCs from RPL

**Figure 10:**
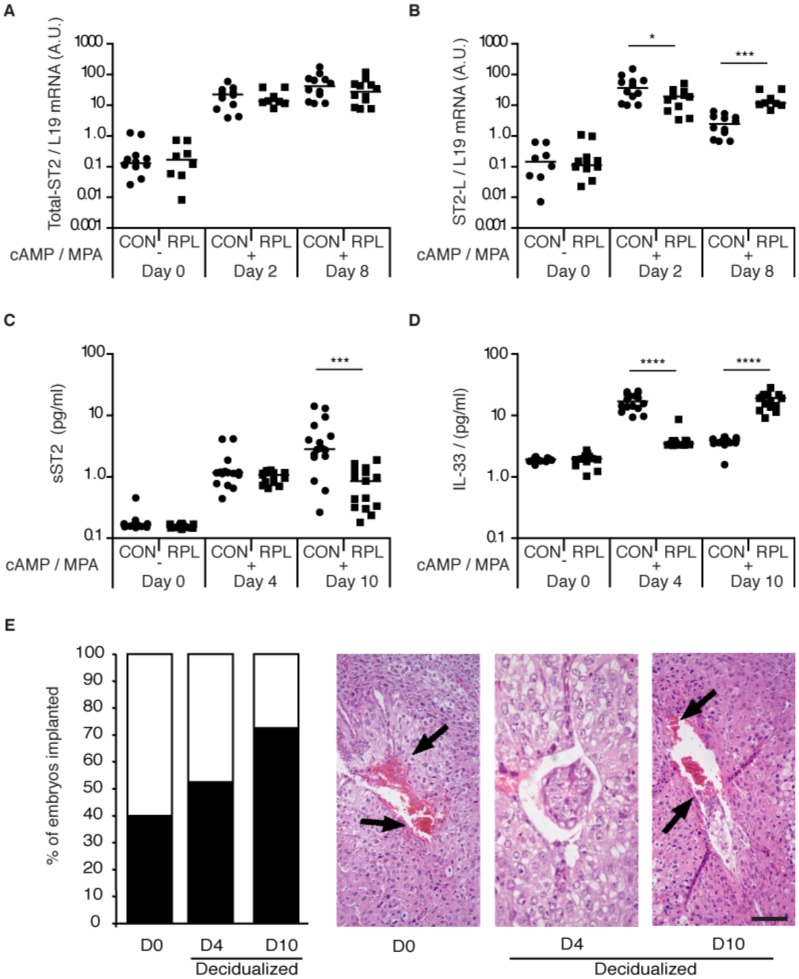
Deregulated IL-33/ST2 activity in decidualizing HESCs from RPL subjects is associated with early pregnancy failure. (A & B) Primary cultures, passaged once, from control (n = 12) and RPL (n = 8) subjects were treated with 8-Br-cAMP and MPA (cAMP/MPA) for 2 or 8 days. Total ST2 and ST2L transcript levels, normalized to L19 mRNA, were measured and expressed in arbitrary units (A.U.). Horizontal bars indicate the median expression in each group. Note the logarithmic y-axes. *P<0.05; ***P<0.001. (C & D) Additional cultures from control (n = 15) and RPL (n = 16) subjects were treated with 8-Br-cAMP and MPA (cAMP/MPA). The medium was changed every 48 hours. Accumulated levels of secreted sST2 and IL-33 were measured at 4 and 8 days of treatment. Horizontal bars indicate the median expression in each group. Note the logarithmic y-axes. ***P<0.001; ****P<0.0001. (E) The murine implantation model was used to examine the consequences of a prolonged and disordered pro-inflammatory response in decidualizing HESCs from RPL patients. In each treatment group, a total of 40 embryos were transferred in 4 animals. The number of implantation sites (left panel) in the right horn was determined 72 h after blastocyst transfer (equivalent of 6.5 d.p.c.). The right panel shows a representative implantation sites. Note the hemorrhagic resorption (arrows) and poor decidualization response in the D0 and D10 HESC supernatant treatment groups. (Scale bar: 100 µm).

The decidual response is very divergent between primary HESC cultures established from RPL and non-RPL subjects [13], [37]. To determine whether the aberrant decidual response associated with RPL also encompasses the IL-33/ST2L pathway, we established primary HESC cultures from 8 RPL patients and 12 controls (Table 3). The cultures were passaged once, grown to confluency, and then decidualized with 8-br-cAMP and MPA for either 2 or 8 days. As expected, total ST2 transcript levels increased markedly in differentiating HESCs and levels were comparable between RPL and control groups (Fig. 10A). In contrast, the induction of ST2L transcripts peaked at 2 days of treatment with 8-br-cAMP and MPA in control samples (*P*<0.05). This bi-phasic pattern of induction was absent in RPL samples and ST2L mRNA levels remained elevated in cells decidualized for 8 days (*P*<0.001) (Fig. 10B). Secreted sST2 and IL-33 levels were measured in additional RPL (n  = 15) and non-RPL (n  = 16) primary cultures decidualized for 4 or 10 days. The media was changed every 48 h. Interestingly, primary cultures from RPL patients secreted significantly lower levels of sST2 upon prolonged (day 10) decidualization (*P*<0.001) (Fig. 10C). Even more striking was the difference in IL-33 secretion between the two clinical groups. In control cultures, approximately 10-times more IL-33 was secreted by 4 days compared to 10 days of decidualization whereas the reverse was true for RPL cultures (*P*<0.0001) (Fig. 10D).

**Table 3:** Patient characteristics and culture details.

qRT-PCR (Fig 10 A & B)	Control	RPL
Number of patients	12	8
Age (years)	33±4.9	32.2±3.6
Live births	0.3±1	0.4±0.7
Miscarriages	0.1±0.3	5.6±2.2*
LMP	17±5.6	16.2±4.5
Days in culture	16±5.1	15.4±2.3
ELISA (Fig 10 C & D)	Control	RPL
Number of patients	16	15
Age (years)	34±3.1	32.3±6.6
Live births	0.4±0.9	0.2±0.4
Miscarriages	0	7.0±4.7*
LMP	17.6±7.1	20.8±5.3
Days in culture	15±5.3	15.3±4.6

The control group consisted of patients with male factor,peritoneal/tubal or unexplained infertility. The data presented are means ± standard deviation. LMP =  last menstrual period. *indicates *P<*0.001.

Next we examined if the prolonged pro-inflammatory response in decidualizing HESCs from RPL patients impacted on embryo implantation in the mouse uterus. Out of 40 embryos transferred in each group, 16 (40%) and 21 (52%) implanted after uterine exposure to secreted factors from undifferentiated cells and cells decidualized for 4 day, respectively (Fig. 10E). This was significantly lower when compared to the 29 (72.5%) embryos that implanted following flushing with conditioned medium from primary RPL cultures decidualized for 10 days (*P*<0.05). Interestingly, although embryo implantation was least impaired in response to exposure to signals of HESCs decidualized for 10 days, approximately 90% of pregnancies displayed defects in the feto-maternal interface 3 days later (Table 4). Thus, while paracrine signals from RPL cultures decidualized for 10 days fail to render the endometrium refractory, the out-of-phase uterine environment at implantation seems to compromise the subsequent pregnancy trajectory.

**Table 4:** Analysis of murine implantation sites after exposure to soluble factors from HESCs.

Control subjects: Number of implantation sites examined	Normal (%)	Abnormal (%)
D0 (n = 9)	55.6	44.4
D4 (n = 28)	89.3	10.7
D10 (n = 26)	26.9	73.1
**RPL subjects: Number of implantation sites examined**	**Normal (%)**	**Abnormal (%)**
D0 (n = 28)	14.3	85.7***
D4 (n = 18)	72.2	27.8*
D10 (n = 18)	11.1	88.9

Abnormal implantation sites were defined as those that had excessive focal bleeding, immune cell infiltration, obviously small for gestational age, or twin implantation. The incidence of abnormal implantation sites in the mouse uterus was significantly higher following exposure to soluble factors secreted by HESCs at D0 and D4 from RPL compared to control subjects. ***indicates *P<*0.001;*indicates *P<*0.05.

## Discussion

Gene ablation studies in mice have been instrumental in identifying key implantation regulators. Within this growing network of genes many encode secreted factors, including growth factors (e.g. Hb-egf), cytokines (e.g. Lif, Il-1β, Prok1, and Il-11) and various morphogens (e.g. Ihh, Wnt4, Bmp2) [1], [38], [39]. These factors not only signal to the pre-implantation embryo but also establish paracrine gradients that control differentiation of specific cell types in a distinct temporal-spatial order. For example, Ihh signaling, emanating from the receptive epithelium and acting on the underlying stroma, is indispensable for both implantation and decidualization [40], [41]. Mesenchymal-epithelial signaling is equally important for implantation. This was elegantly illustrated by a recent study demonstrating that progesterone, acting on its receptor in stromal cells, induces the basic helix-loop-helix transcription factor Hand2 [42]. By suppressing the production of fibroblast growth factors that mediate the mitogenic effects of estrogen on the epithelium, Hand2 in stromal cells allows neighboring epithelial cells to exit the cell cycle and to enter a differentiation pathway leading to the window of implantation [42]. Thus, sequential activation of bidirectional paracrine signal cascades between the stromal and epithelial compartments in murine endometrium triggers the receptive endometrial phenotype and couples it to the decidual response upon embryo implantation [2], [43].

Decidualization of the stromal compartment in the human endometrium is equally tightly controlled. However, this differentiation process is no longer under the primary control of an implanting embryo, as is the case in most mammals. Instead, it is triggered during the mid-secretory phase of each cycle in response to local factors, such as prostaglandin E2, that activate the cAMP pathway [5]. Once initiated, the phenotype is maintained as long as progesterone levels remain high. We now show that cAMP signaling drives the expression of IL-33 in HESCs whereas progestins upregulate ST2 transcripts. The presence of IL-33 in human endometrium is not unexpected as this cytokine is abundantly expressed in various mucosal and epithelial barrier tissues [28], [44]. Its nuclear expression combined with the absence of a peptide signal for export led to the notion that IL-33 is released primarily upon tissue injury or infection and, through binding to ST2L on immune cells, responsible for initiating tissue inflammation [28], [45]. Unexpectedly, we found that decidualizing HESCs actively secrete IL-33. Notably, recent studies showed that viable cells can release IL-33, for example in response to cellular stretch or mechanical strain [46]. It is therefore not inconceivable that the profound changes in cell shape and actin dynamics upon decidualization trigger nuclear export and secretion of IL-33 via an unconventional pathway. Binding of IL-33 to ST2L on the cell surface of differentiating HESCs establishes an autocrine pathway that drives an acute phase response, characterized by coordinated induction of interleukins, chemokines, C-reactive protein and several other inflammatory mediators. STL2 activity in decidualizing cells is intrinsically self-limiting as IL-33 combined with continuous progestin signaling decreases transmembrane receptor levels and stimulates the secretion of the anti-inflammatory decoy receptor sST2. Transition from the pro- to anti-inflammatory phase of the decidual process is further characterized by suppression of many pro-inflammatory genes below basal levels in undifferentiated stromal cells and the induction of the Th2 cytokines IL-5 and IL-10.

Several lines of evidence indicated that activation of the IL-33/ST2 pathway in HESCs is critical for a successful pregnancy. First, only during the pro-inflammatory phase did differentiating HESC secrete factors that heightened the expression of murine receptivity genes, enabling efficient implantation of transferred mouse blastocysts. Second, we found that implantation in the mouse uterus was actively impaired upon exposure to signals from undifferentiated HESCs, from decidualizing cells in the anti-inflammatory phase, and from decidualizing cells first transfected with IL-33 siRNA. Importantly, while some embryos did implant under these conditions, a high proportion of the resultant implantation sites exhibited pathological features, including focal bleeding and excessive immune cell infiltration. Notably, mice deficient in either ST2 [47] or IL-33 are reportedly fertile (personal communication with Dr A McKenzie, University of Cambridge), although early pregnancy events have to our knowledge not yet been investigated in these animal models. While speculative, this may relate to interspecies differences in maternal versus embryonic control of the decidual process or point towards a degree of redundancy in the primary trigger of the pro-implantation inflammatory response. Third, we showed that sequential activation of the IL-33/ST2L/sST2 axis is deregulated in primary HESCs from RPL subjects and likely to cause a prolonged window of implantation. Again, many implantation sites showed pathological features, emphasizing that the peri-implantation milieu has profound consequences for the subsequent developmental trajectory of the conceptus. Thus, a prolonged and disordered auto-inflammatory response in resident stromal cells seems to cause considerable collateral damage, resulting in bleeding, excessive recruitment of local immune cells, and subsequent fetal demise. In keeping with our findings, aberrant signaling from the IL-33/ST2L/sST2 axis was associated with late pregnancy complications such as pre-eclampsia [48] and miscarriage [49].

RPL, here defined as 3 or more consecutive miscarriages, is a prevalent disorder that affects 1–2% of couples [50], [51]. It is a cause of considerable physical and psychological morbidity and associated with increased likelihood of obstetric complications and adverse perinatal outcome in a subsequent ongoing pregnancy [51]. Our findings confirm that the cellular response to identical differentiation signals is profoundly divergent between primary cultures established from RPL and control subjects. Further, the data provide novel insights into the nature and pathological consequences of aberrant decidualization, although the underlying molecular mechanism requires further elucidation. Emerging evidence indicates that profound changes in chromatin structure, exemplified by genome-wide redistribution of transcriptionally repressive and permissive histone marks, precede the expression of a decidual phenotype in HESCs [52]. Hence, it is possible that the primary defect in RPL may lie in epigenetic programming of endometrial stromal cells, or their bone marrow-derived stem/progenitor cells [53].

In summary, by restricting the window of implantation, the continuously changing endometrial environment is aligned to meet the requirements of an implanting blastocyst. Prolonged or unfettered endometrial receptivity carries an obvious risk of implantation of developmentally delayed or compromised embryos [13]. Further, asynchrony between endometrial and embryo development in early pregnancy may trigger a spectrum of pathological events, leading to miscarriage or predispose for obstetrical complications associated with defective placentation, such as pre-eclampsia and fetal growth restriction [12], [14]. Our findings show that uterine receptivity is controlled by the activation of autoregulatory feedback loops in decidualizing stromal cells underlying the luminal epithelium that trigger the sequential expression of pro- and anti-inflammatory gene networks. While our observations suggest that stromal cells can exert this function independently of local immune cells, complementation experiments are needed to define the regulatory roles of other endometrial cell types during the implantation process. We also provided evidence that failure of differentiating HESCs to transit from a pro-receptive to a post-implantation phenotype predisposes for subsequent pregnancy failure. Clinically, the IL-33/ST2 pathway is considered a major novel target for therapeutic interventions across a range of diseases, including Alzheimer's disease [54], helminth infections [55], cardiovascular disease [56], obesity [57], asthma [58] and other autoimmune disorders [59], [60]. Our findings suggest that targeting the same pathway in the uterus is a promising strategy to regulate endometrial receptivity.

## Supporting Information

Figure S1 **Schematic representation.** ST2 primer pairs used to amplify all Total-ST2 transcripts (T-ST2) or the transmembrane ST2L (TM-ST2) transcripts.
(TIFF)

Figure S2 **Il-33 knockdown in HESCs decidualized for 2 days triggers an inflammatory response akin to that seen in cells decidualized for 8 days.** (*A*) Number of transcripts significantly up- or down-regulated in each of the indicated categories upon decidualization for 2 or 8 days (D2 and D8, respectively) compared to undifferentiated cells. (*B*) Number of transcripts significantly up- or down-regulated in each of the indicated categories after 2 days of decidualization of primary HESCs cultures transfected with either non-targeting (NT) or IL-33 siRNA (si IL-33).
(TIF)
