# Supplementary figures and images for: Disordered IL-33/ST2 Activation in Decidualizing Stromal Cells Prolongs Uterine Receptivity in Women with Recurrent Pregnancy Loss
Source: PLoS One. 2012 Dec 27;7(12):e52252. doi: 10.1371/journal.pone.0052252 (PMC3531406; doi:10.1371/journal.pone.0052252)

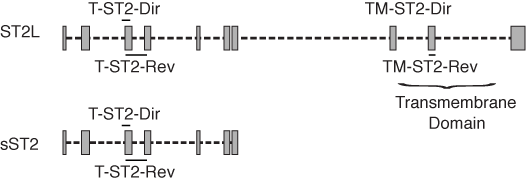

Supplement: Figure S1 — Schematic representation. ST2 primer pairs used to amplify all Total-ST2 transcripts (T-ST2) or the transmembrane ST2L (TM-ST2) transcripts. (TIFF) [file pone.0052252.s001.tiff]

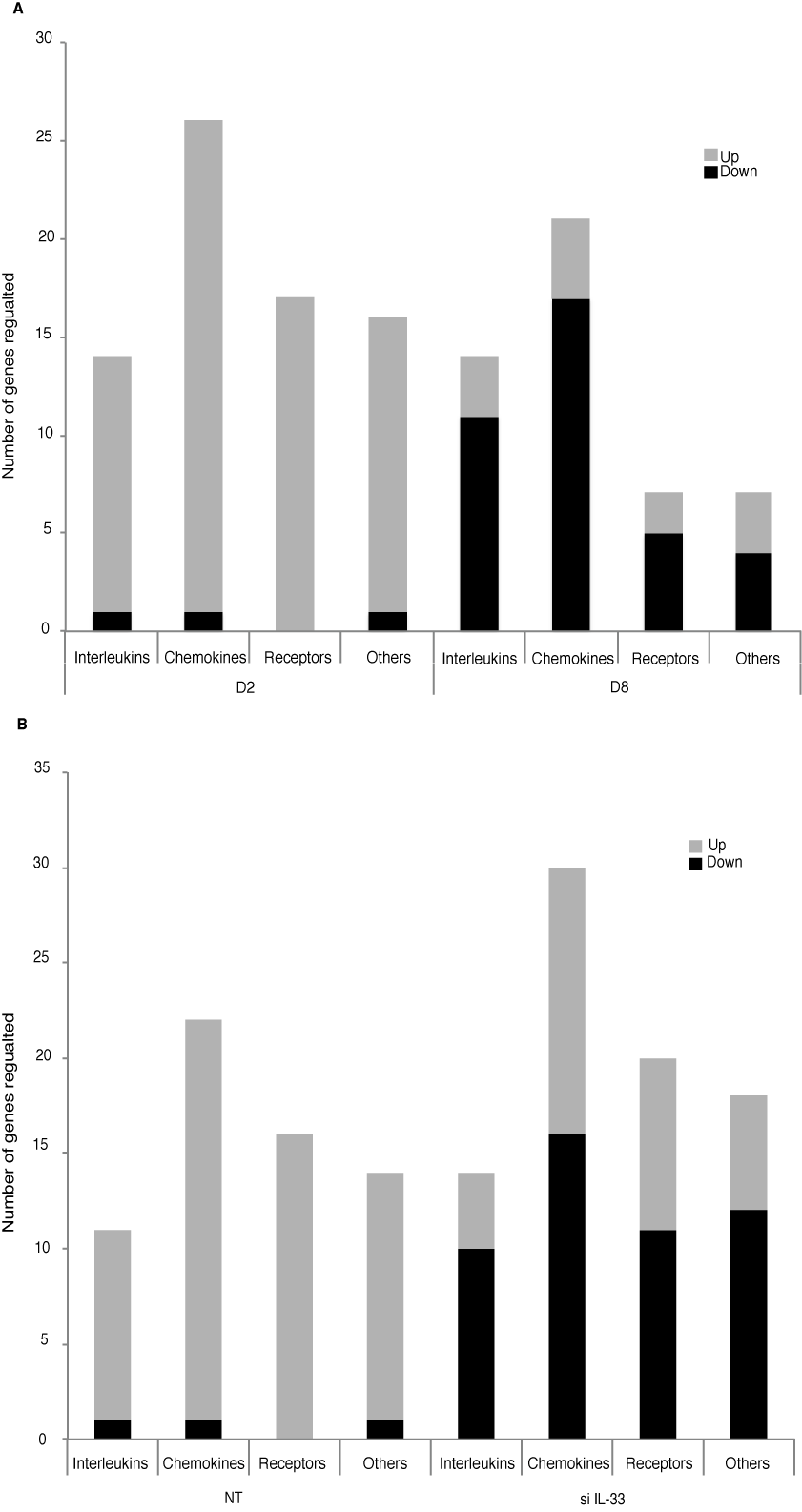

Supplement: Figure S2 — Il-33 knockdown in HESCs decidualized for 2 days triggers an inflammatory response akin to that seen in cells decidualized for 8 days. (A) Number of transcripts significantly up- or down-regulated in each of the indicated categories upon decidualization for 2 or 8 days (D2 and D8, respectively) compared to undifferentiated cells. (B) Number of transcripts significantly up- or down-regulated in each of the indicated categories after 2 days of decidualization of primary HESCs cultures transfected with either non-targeting (NT) or IL-33 siRNA (si IL-33). (TIF) [file pone.0052252.s002.tif]
